# Sociodemographic, Health, and Lifestyle-Related Characteristics Associated With the Commencement and Completion of a Web-Based Lifestyle Educational Program for People With Multiple Sclerosis: Randomized Controlled Trial

**DOI:** 10.2196/58253

**Published:** 2024-08-28

**Authors:** Jeanette Reece, Maggie Yu, William Bevens, Steve Simpson-Yap, Rebekah Davenport, George Jelinek, Sandra Neate

**Affiliations:** 1 Neuroepidemiology Unit Melbourne School of Population and Global Health The University of Melbourne Melbourne Australia; 2 Institute for Clinical and Translational Science University of California Irvine, CA United States; 3 The Florey Institute of Neuroscience and Mental Health The University of Melbourne Melbourne Australia; 4 Menzies Institute for Medical Research University of Tasmania Hobart Australia; 5 Melbourne School of Psychological Sciences The University of Melbourne Melbourne Australia

**Keywords:** multiple sclerosis, web-based education, lifestyle, randomized controlled trial, engagement, completion

## Abstract

**Background:**

Digital health interventions increase access to multiple sclerosis (MS)–related knowledge for people living with MS; however, our understanding of factors associated with engagement in web-based learning is limited.

**Objective:**

This study aims to examine associations between participant sociodemographic, health, and lifestyle-related characteristics and the commencement and completion of the Multiple Sclerosis Online Course (MSOC) in a randomized controlled trial (RCT).

**Methods:**

An intervention course was developed based on the Overcoming MS Program—an evidence-based lifestyle modification program for MS, and a standard care course was developed based on international MS website information. An RCT was conducted to compare the effectiveness of the intervention course versus the standard care course in improving health outcomes in people living with MS. Participant data were collected from a baseline survey. Associations between baseline participant characteristics and MSOC commencement and completion, respectively, were assessed using multivariate log-binomial regression.

**Results:**

Overall, 1893 participants enrolled in the RCT, and 45.27% (n=857) completed the baseline survey: 23.5% (n=444) in the intervention course and 21.8% (n=413) in the standard care course. Of these 857 participants, 631 (73.6%) commenced the standard care course or intervention course, and 49.1% (218/444) and 54.2% (224/413) completed the intervention course and standard care course, respectively. University education, partnered relationship status, and higher mental and physical quality of life were associated with 19%, 12%, 20%, and 22% higher rates of course commencement, respectively. Clinically significant fatigue was associated with a 10% reduction in the likelihood of commencement. Strongest associations with intervention course completion included middle and older adulthood, male sex, fatigue, and preexisting adherence to a diet program, with 96%, 27%, 24%, and 19% higher rates of completion observed, respectively, whereas higher self-efficacy was associated with up to 35% lower intervention course completion. Associations with standard care course completion included practicing meditation (20% higher completion), whereas employment was associated with 22% lower completion.

**Conclusions:**

Sociodemographic and clinical factors, as well as lifestyle-related factors, were important factors in MSOC commencement and completion. These data may help guide the design and enhancement of digital health interventions tailored for people living with MS.

**Trial Registration:**

Australian New Zealand Clinical Trials Registry ACTRN12621001605886; https://tinyurl.com/2vyve9p9

**International Registered Report Identifier (IRRID):**

RR2-10.1186/s12883-023-03298-0

## Introduction

### Background

There is a growing body of evidence supporting the role of modification of lifestyle-related risk factors on enhancing health outcomes in people living with multiple sclerosis (MS) [1-3]. Subsequently, the management of MS has evolved over recent years, with people living with MS placing a greater emphasis on self-management of MS and seeking lifestyle-related information as an integral part of maintaining and improving their health [4].

MS is a progressive autoimmune condition manifesting in sensory, motor, and cognitive dysfunction, the cardinal clinical elements of which are disability progression and relapse (as defined by a clinical exacerbation of symptoms involving the development of 1 or more new MS symptoms or worsening of existing symptoms lasting >48 hours, with changes in symptoms not due to extraneous conditions such as heat or illness, eg, respiratory or urinary tract infections) [5]. Modification of lifestyle-related risk factors in MS has been associated with a lower rate of relapse, reduced fatigue, disability progression, and depressive symptoms and higher quality of life (QoL) [6-12]. A program for the modification of lifestyle-related risk factors in MS, the Overcoming Multiple Sclerosis (OMS) Program [13], provides recommendations for a plant-based whole food plus seafood diet low in saturated fat, regular physical activity, vitamin D and omega-3 fatty acid supplementation, smoking cessation, and stress reduction. These recommendations have been delivered as a face-to-face educational intervention with demonstrated effectiveness, including adherence to lifestyle recommendations (a healthy diet, meditation, and vitamin D and omega-3 supplementation) 3 years after attending the retreat and associations with increased QoL in both the short (1 year) and medium (5 years) term [14-16]. However, the reliance on face-to-face delivery hinders the scalability and accessibility of the educational intervention for all people living with MS. Web-based lifestyle modification programs offer a scalable solution, overcoming commonly documented barriers such as mobility to travel outside of the home and additional financial costs [17].

Several web-based lifestyle modification interventions have been developed for people living with MS to increase MS-related knowledge [18], build resilience [19], and facilitate lifestyle changes [20]. Many have demonstrable effectiveness, such as reduced depression and fatigue [21,22]. Furthermore, interventions have been shown to improve walking ability; reduce overall neurological disability [20]; and improve depression, anxiety, and sleep [23]. Recently, the *Understanding* *MS* massive open online course (MOOC) led to increases in MS-related knowledge and health literacy [18] and lifestyle changes (diet, physical activity, and vitamin D supplementation) in people living with MS [24]. More recent studies have examined the usability of web-based education interventions, with an intervention aimed specifically at facilitating multimodal behavior change in people living with MS with moderate to severe disability found to be both practical and acceptable [25]. Similarly, a web-based nutrition education program demonstrated good acceptability using a co-design development model to incorporate the needs of people living with MS themselves [26]. As such, this nascent field of digital health for MS-related lifestyle modification has emerged as an important potential tool to support people living with MS.

Despite the potential value of digital interventions, engagement with web-based interventions compared to face-to-face interventions is generally low [27]. Course completion rates vary, with one study demonstrating that as few as 15% to 19% of people living with MS enrolled in digital health programs completed the course [28], whereas other studies have described completion rates that differ from these estimates [29]. While there has been broad implementation and acceptance in the general population, there has been limited exploration of how people living with MS use digital health technologies to support their health and well-being. There is previous evidence suggesting modest variability in the completion of digital health interventions by people living with MS [28,30]; however, factors that affect completion remain unclear. There is other evidence suggesting that the use of digital technologies varies significantly by sociodemographic factors and depending on what the technology is used for [31]. We also previously reported that sourcing lifestyle information on the web is precarious for people living with MS because critically appraising information can be difficult without professional assistance from trustworthy sources [32]. In this context, programmatic and structured web-based educational interventions developed by medical professionals and researchers may be an effective way to engage people living with MS with lifestyle modification information. An important next step is to explore factors affecting the completion of digital health interventions for people living with MS to ensure that future developments can meet their needs and deliver education effectively.

### Objectives

This study aimed to identify the characteristics of people living with MS associated with the commencement and completion of the Multiple Sclerosis Online Course (MSOC) by examining sociodemographic, health, and lifestyle-related characteristics of participants enrolled in a randomized controlled trial (RCT) [33]. Study findings have the potential to increase our understanding of the barriers and enablers of the completion of web-based, lifestyle-related interventions by people living with MS, providing a basis for the development of future digital interventions.

## Methods

### Ethical Considerations

This ancillary RCT is a CONSORT-R (Consolidated Standards of Reporting Trials–Routine)–compliant RCT (Multimedia Appendix 1). The protocol for the RCT was reviewed by the Australian New Zealand Clinical Trials Registry and approved on November 25, 2021 (ACTRN12621001605886). The study was reviewed by the University of Melbourne Human Research Ethics Committee and approved on November 2, 2021 (22140). Participants were invited via web-based platforms to participate in the RCT and did not receive any monetary compensation for participating in the RCT. Participants were provided with a participant information statement, and written informed consent was obtained from all participants for their data to be used for research purposes before inclusion in the RCT. The signed consent form outlined that the confidentiality of their data would be ensured as per safeguard legal requirements. For analyses and reporting, all participant data were stored in a reidentifiable format to ensure participants’ privacy and confidentiality at the University of Melbourne in the form of password-protected computer databases, and only the listed investigators had access to the data.

### MSOC RCT Study Design

The primary aims of the ancillary RCT (the MSOC effectiveness RCT) included examining (1) changes in the health-related QoL of people living with MS from baseline to the 6-, 12-, and 30-month follow-ups (primary outcome); and (2) changes in other health outcomes (depression, anxiety, fatigue, and disability) between baseline and the 6-, 12-, and 30-month follow-ups (secondary outcome) [33]. The secondary aims included examining changes in lifestyle from baseline to the 6-, 12-, and 30-month follow-ups. This study represents an additional analysis of baseline data collected as part of the flagship RCT and aimed to examine factors associated with commencement and completion of the MSOC. All baseline data were collected from June 2022 to July 2023. Data analysis was performed at the completion of all baseline data collection from November 2023 to December 2023.

People living with MS were invited to participate in the RCT and complete the MSOC. Data from the following participants were excluded from all analyses: (1) participants experiencing any serious comorbid chronic illness or neurological illness or injury other than MS that would threaten regular participation or significantly affect the outcome measures in its own right, such as motor neuron disease or stroke, as determined by the study investigators; and (2) participants currently taking part in another RCT.

While we did not notify participants that their data would be used specifically for this study, participants were aware that we were examining factors associated with commencement and completion of the web-based intervention outlined in the postcourse evaluation questionnaire sent to all participants upon completion of the course as per the study protocol [33].

### MSOC Effectiveness RCT Study Design

The MSOC effectiveness RCT has previously been described in detail [33]. In brief, the RCT aimed to assess the effectiveness of a 6-week intervention course in improving QoL and health outcomes in people living with MS compared with a 6-week standard care course. The intervention course modules provided content adapted from the OMS evidence-based lifestyle modification program (Multimedia Appendix 2) [13]. The standard care course contained standard health recommendations sourced from international public MS society websites that aimed to reflect standard information provided by health care practitioners and MS societies.

Both courses comprised 7 modules, commencing with a *Welcome to the MSOC Study* module containing a plain-language statement and baseline survey followed by five educational modules: (1) *Introduction*, (2) *Eat well*, (3) *Sunlight and vitamin D*, (4) *Exercise*, (5) *Meditation and the mind-body connection*, (6) *Medication and family prevention*, and (7) *Conclusion*. In total, 2 modules were released each week over a 4-week period, and a further 2 weeks were provided for course completion. The feasibility of the intervention course and standard care course at delivering educational content to people living with MS has been previously demonstrated [34].

The modules of the intervention course and standard care course mirrored one another in format and style of delivery of content in terms of a combination of videos, animations, visuals, and discussions from presenters. Key differences included the intervention course’s focus on specific lifestyle recommendations supplemented by video discussions, illustrations, and web resources for in-depth exploration. For instance, the intervention course advised a specific plant-based whole food diet plus seafood with very low saturated fat (<20 g/d) excluding dairy, meat, and palm and coconut oil, complemented with recipes and video discussions on selecting ingredients and adherence tips. Furthermore, for daily meditation, the intervention course offered practical video guidelines for practices of ≥30 minutes per day. In contrast, the standard care course provided general advice without detailed guidance; that is, the standard care course offered broader advice, recommending a balanced diet based on national guidelines and mentioning meditation without providing solid evidence of its efficacy in MS management.

### Participants

Participants were recruited on the web via peer support Facebook groups worldwide and MS societies in Australia, Canada, Ireland, New Zealand, and the United Kingdom. Recruitment flyers were posted on Facebook, Twitter, and Instagram. Interested participants completed 2 eligibility questions at the study website confirming that they were aged ≥18 years and had received a physician-confirmed diagnosis of MS. Participants were required to speak English to be able to understand the course content.

Eligible persons were sent a link to set up an account and log in to the course platform. Participants were then allocated to the intervention course or standard care course at a 1:1 ratio using simple randomization. Participants were also requested to complete a 166-question baseline survey on sociodemographic, health, and lifestyle-related factors. If participants did not complete the baseline survey, they were sent 2 email reminders to complete it, but this did not prevent access to the web-based course, which was provided to all enrolled participants regardless of whether they completed the baseline survey. However, only data from participants who completed the baseline survey were included in this study to address study objectives. This comprised data from 857 participant baseline surveys collected during the 5 rounds of RCT recruitment that ran between June 23, 2022, and September 4, 2023. Each of the 5 recruitment rounds used the same strategy to recruit participants; that is, recruitment for the RCT involved advertising on international MS websites and MS-related social media sites such as Facebook and Instagram.

### Sociodemographic Characteristics

Data on age, sex, employment, level of education, and marital status were collected. Specifically, we queried the highest level of education (no formal schooling, primary school, secondary school, vocational training, bachelor’s degree, or postgraduate degree), marital status (married; cohabitating or partnered; separated, widowed, or divorced; or single), and current work status (10 categories ranging from working full time to retired due to medical reasons or disability and work status not clearly defined). Perceived social support was measured using the 12-item Multidimensional Scale of Perceived Social Support survey [35]. A summary Multidimensional Scale of Perceived Social Support score was calculated, with higher scores indicating higher perceived support.

### Health Characteristics

Height (centimeters or inches) and weight (kilograms or pounds) were used to calculate BMI, categorized as underweight (<18.5 kg/m^2^), normal (18.5-24.9 kg/m^2^), overweight (25.0-29.9 kg/m^2^), and obese (≥30.0 kg/m^2^) as per World Health Organization guidelines [36]. MS type was categorized into nonprogressive (benign or relapsing-remitting MS) and progressive (primary progressive, secondary progressive, or progressive-relapsing MS). MS duration was calculated using the year of diagnosis and baseline survey date. The number of treated comorbidities was queried using the Self-Administered Comorbidity Questionnaire [37] and categorized as 0 and ≥1. Ongoing symptoms from relapse within ≤30 days were queried (yes or no). The use of disease-modifying therapies (DMTs) was queried (yes or no), and if yes, the type of DMT was queried.

Disability was assessed using Patient-Determined Disease Steps scale scored ordinally from 0 (normal) to 8 (bed bound) [38,39] and categorized into none or mild (0-2), moderate (3-5), and severe (6-8) disability. Fatigue was measured using the 9-item Fatigue Severity Scale, with a mean score of >5 indicating clinically significant fatigue [39,40]. QoL was measured using the Multiple Sclerosis Quality of Life-54, and 2 composite scores for mental and physical QoL and 12 subdomains scored from 0 (low) to 100 (high) were calculated [41].

Self-efficacy was measured using the 6-item University of Washington Self-Efficacy Scale (UWSES) [42]. The UWSES is an item response theory–based tool designed to measure disability management self-efficacy that was originally developed for people living with MS but has also been validated for adults living with other chronic health conditions. In particular, the UWSES queries people living with MS to assess whether they believe they can manage their health condition or disability, for instance, whether they are able to keep their health condition or disability from being the center of their life or interfering with how they deal with unexpected events or social interactions. As no clinically significant cutoff for self-efficacy is reported, a summary score was calculated, with higher UWSES scores indicating greater self-efficacy.

Anxiety and depressive symptoms were measured using the 14-item self-report Hospital Anxiety and Depression Scale [43]. The Hospital Anxiety and Depression Scale includes a 2-factor structure—depression and anxiety—each measured using 7 items. Scores range from 0 (no symptoms) to 21 (most severe symptoms), with scores of 0 to 7 considered “normal,” scores of 8 to 10 considered “borderline anxiety or depression,” and scores of 11 to 21 considered moderate to severe anxiety or depression.

### Lifestyle-Related Characteristics

Previous participation in another lifestyle course or intervention (no or yes) and undertaking a particular diet program for MS (no or yes) and, if yes, the type of lifestyle course or intervention or diet were queried. Diet quality was measured using the modified Diet Habits Questionnaire (DHQ), previously validated in people living with MS over a 24-hour recall period [44], but excluding questions on salt and alcohol [45,46]. Responses were scored and summated. Total DHQ scores were estimated as a score out of 100. Total DHQ scores range between 20 and 100, with higher DHQ scores indicating a better quality of diet. Total scores were categorized into quartiles as in the MS-related study by Kirkland et al [47], with scores of >80 indicative of a “healthier” overall reported dietary intake. Physical activity was measured using the International Physical Activity Questionnaire (IPAQ) and categorized as inactive, minimally active, and active as per IPAQ guidelines [48]. The IPAQ has been used to evaluate physical activity in people living with MS in previous studies [49], including digital interventions aimed at increasing physical activity [50]. Frequency and quantity of alcohol consumption were queried and categorized into tertiles (none [no alcohol intake], limited [≤1 standard drink per day for female individuals and ≤2 standard drinks per day for male individuals], and heavy drinking [>1 standard drink per day for female individuals and >2 standard drinks per day for male individuals]) as in previous MS-related studies [3,10]. Participants were queried as to whether they consumed meat, dairy, and vitamin D and omega-3 supplements (no or yes). Current smoking status was dichotomized as current versus never or ex-smoker. Formal meditation practice (eg, sitting meditation) in the past week was queried (no or yes).

### Data Collection

This study is an additional analysis of quantitative data collected at baseline before course commencement. While not part of this study, the ancillary RCT will collect quantitative exposure and outcome data at 6, 12, and 30 months following course completion to examine the primary and secondary aims as per the study protocol [33]. Qualitative interviewing of approximately 40 course completers across both study arms will also be performed at 1 and 12 months after course completion to develop a deeper understanding of participants’ experiences of the course and any impacts of the course.

### Outcome Variables

In total, 2 measures of course engagement were examined: course commencement and course completion. We examined course commencement as a proxy for measuring course engagement by evaluating the relationship between participants being interested in the course (ie, signing up to undertake the course) and following through to commence the course. Course completion is also a commonly used metric to measure acceptability of web-based interventions [28].

Course commencement was defined as completion of at least the introductory module (module ≥1). For commencement analyses, course commencers (completers of module ≥1) were compared with course noncommencers (noncompleters of module 1). Intervention course and standard care course commencement was examined collectively due to the similarity of module 1 in both courses.

Course completion was defined as the completion of modules 1 to 6 as module 7 comprised a closing session and did not provide lifestyle-related information. For completion analyses, among course commencers (completers of module ≥1), course completers (completers of modules 1-6) were compared with course noncompleters (completers of module 1 but not module 6).

### Data Analysis

Associations between participants’ sociodemographic, health, and lifestyle-related characteristics and course commencement were examined. In addition, among course commencers, associations between participant characteristics and course completion stratified by the standard care course and intervention course study arms were examined.

Multivariate log-binomial regression was used to examine associations, with results presented as prevalence ratios and 95% CIs. Models were adjusted for age, sex, educational level, MS type, experience of ongoing symptoms from recent relapse, physical comorbidities, disability, use of DMTs, and participation in another lifestyle course or diet program at baseline. Model covariates were selected based on significant independent associations with exposure and outcome terms in the current data set in univariate analyses, as well as from review of relevant studies in the literature [3,6]. To ensure the validity of the regression models, we conducted collinearity diagnostics, which revealed no evidence of multicollinearity among the examined variables. This was confirmed through variance inflation factor and tolerance statistics. All statistical analyses were performed using Stata (version 16.0; StataCorp). Statistical significance was defined as *P*<.05.

## Results

### Baseline Participant Characteristics

Participants were of a mean age of 47.0 (SD 11.7) years; 87.7% (752/857) were female, 49.5% (424/857) had underweight or normal BMI, 65.6% (562/857) were university educated, 55% (471/857) were employed, and 69.8% (598/857) were married or partnered (Table 1).

Participants resided in 53 countries, with most participants residing in North America (286/857, 33.4%), Australia or New Zealand (236/857, 27.5%), and the United Kingdom (113/857, 13.2%) and 25.9% (222/857) residing in 48 other countries (Multimedia Appendix 3).

Most participants (614/857, 71.6%) reported having nonprogressive MS; 55.4% (475/857) reported clinically significant fatigue; and 17.2% (147/857) and 31.6% (271/857) reported symptoms of severe depression and anxiety, respectively (Table 1). The mean duration since MS diagnosis was 9.4 (SD 9.1) years. In total, 52.4% (449/857) of participants had none or mild disability, and 56.7% (486/857) reported ³1 comorbidity. A total of 67.8% (581/857) were taking DMTs, with ocrelizumab (164/857, 19.1%), natalizumab (56/857, 6.5%), and ofatumumab (53/857, 6.2%) being the most commonly reported (Multimedia Appendix 4).

Regarding lifestyle-related characteristics, 26.5% (227/857) of participants were following a lifestyle program or undertaking some lifestyle modifications (OMS: 68/857, 7.9%; exercise: 43/857, 5%; stress reduction: 9/857, 1%; other health-related modifications: 9/857, 1%), and 28.1% (241/857) followed a specific diet for MS (OMS: 17/241, 7.1%; gluten free: 12/241, 5%; vegetarian/vegan: 7/241, 2.9%; Mediterranean: 2/241, 0.8%; other MS-related diets [eg, Wahls]: 2/241, 0.8%; other healthy diets [eg, low sugar]: 14/241, 5.8%). A large proportion of participants were taking vitamin D (721/857, 84.1%) and omega-3 (296/857, 34.5%) supplements, 9.3% (80/857) were current smokers, 4.6% (36/857) were heavy drinkers, 28.2% (242/857) practiced meditation, and 19.1% (164/857) engaged in “active” levels of physical activity.

**Table 1:** Descriptive statistics of the study sample (n=857)^a^.

Characteristics			Values
Age (y), mean (SD)			47.0 (11.7)
Sex (female), n (%)			752 (87.7)
**BMI (kg/m^2^), n (%)**			
	Underweight or normal	424 (49.5)	
	Overweight	212 (24.8)	
	Obese	219 (25.6)	
**Country of residence, n (%)**			
	Australia or New Zealand	236 (27.5)	
	United States or Canada	286 (33.4)	
	United Kingdom	113 (13.2)	
	Other	222 (25.9)	
Educational level (university), n (%)			562 (65.6)
Employment status (working), n (%)			471 (55.0)
Marital status (partnered), n (%)			598 (69.8)
MS^b^ type (nonprogressive), n (%)			614 (71.6)
**MS duration (y), n (%)**			
	≤2	245 (28.6)	
	3-6	203 (23.7)	
	7-15	215 (25.1)	
	>15	194 (22.6)	
**Disability (PDDS^c^), n (%)**			
	None or mild	449 (52.4)	
	Moderate	323 (37.7)	
	Severe	85 (9.9)	
**Depression, n (%)**			
	Normal (HADS–Depression^d^ 0-7)	515 (60.2)	
	Borderline (HADS–Depression >7-10)	193 (22.6)	
	Severe (HADS–Depression >10-14)	147 (17.2)	
**Anxiety, n (%)**			
	Normal (HADS–Anxiety^e^ 0-7)	382 (44.6)	
	Borderline (HADS–Anxiety >7-10)	203 (23.7)	
	Severe (HADS–Anxiety >10-14)	271 (31.6)	
Clinically significant fatigue (mean FSS^f^ >5), n (%)			475 (55.4)
Comorbidities (≥1), n (%)			486 (56.7)
Taking DMTs^g^, n (%)			581 (67.8)
Following a diet program for MS, n (%)			241 (28.1)
Participating in a lifestyle program or intervention, n (%)			227 (26.5)
Vitamin D supplements (any), n (%)			721 (84.1)
Omega-3 supplements (any), n (%)			296 (34.5)
**Smoking,** **n (%)**			
	Never smoker	482 (56.2)	
	Ex-smoker	295 (34.4)	
	Current smoker	80 (9.3)	
**Alcohol consumption, n (%)**			
	None	195 (24.7)	
	Limited	527 (66.6)	
	Heavy	36 (4.6)	
Meditation, n (%)			242 (28.2)
**Exercise (IPAQ^h^), n (%)**			
	Inactive	319 (37.2)	
	Minimally active	374 (43.6)	
	Active	164 (19.1)	

^a^444 recruited participants randomized to the intervention course study arm completed the baseline survey, and 413 participants in the standard care course study arm completed the baseline survey.

^b^MS: multiple sclerosis.

^c^PDDS: Patient-Determined Disease Steps.

^d^HADS–Depression: Hospital Anxiety and Depression Scale for symptoms of depression.

^e^HADS–Anxiety: Hospital Anxiety and Depression Scale for symptoms of anxiety.

^f^FSS: Fatigue Severity Scale.

^g^DMT: disease-modifying therapy.

^h^IPAQ: International Physical Activity Questionnaire.

### Course Commencement

**Figure 1:**
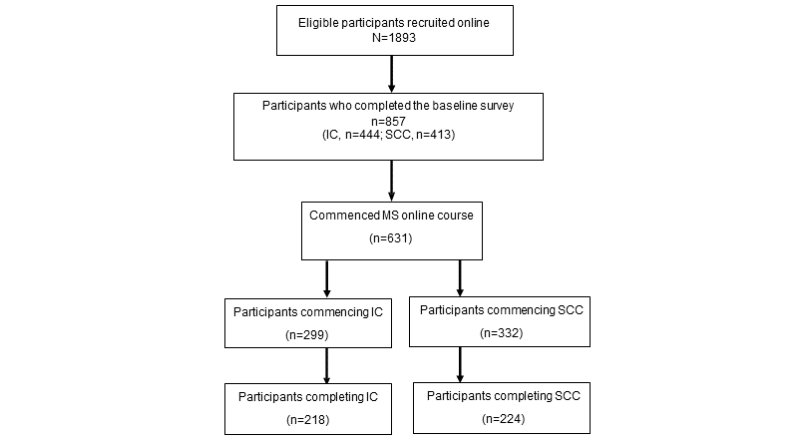
CONSORT (Consolidated Standards of Reporting Trials) flowchart of the study cohort. IC: intervention course; MS: multiple sclerosis; SCC: standard care course.

Of the 1893 participants enrolled in the RCT, 857 (45.27%) completed the baseline survey—444 (23.45%) in the intervention course and 413 (21.82%) in the standard care course. A total of 33.33% (631/1893) of the total number of enrolled participants and 73.6% (631/857) of the participants who completed the baseline survey commenced the MSOC (Figure 1). The proportions of participants who completed the baseline survey and the MSOC were similar across study arms; completion rates in the intervention course were 49.1% (218/444) versus 54.2% (224/413) in the standard care course.

Following multivariate analysis, educational level, marital status, country of residence, mental and physical QoL, and fatigue were associated with MSOC commencement (intervention course and standard care course combined; Table 2).

A university degree or being in a relationship was associated with a 19% (95% CI 7%-32%) and 12% (95% CI 1%-25%) higher likelihood of MSOC commencement, respectively. Participants residing outside Australia or New Zealand, the United States, Canada, or the United Kingdom were 16% (95% CI 4%-26%) less likely to commence the MSOC.

People living with MS with fatigue were 10% (95% CI 1%-18%) less likely to commence the course, whereas people living with MS with mental and physical QoL scores in the top quartiles were 20% (95% CI 4%-38%) and 22% (95% CI 4%-43%) more likely to commence the MSOC, respectively. On the basis of our previous work [10], higher QoL is associated with less clinical severity, better mood, and generally better well-being. Specifically, being in the higher quartiles of categorized QoL indicates that participants with greater health and well-being were approximately 20% more likely to commence the MSOC.

**Table 2:** Associations between sociodemographic, health, and lifestyle-related characteristics and course commencement^a^.

Characteristics				Participants (n=613)^b^, n (%)		Univariate analysis, PR^c^ (95% CI)		Multivariate analysis, aPR^d^ (95% CI)
**Sociodemographics^e^**								
	**Age (y)**							
		≤35	108 (17.1)		1.00 (reference)		1.00 (reference)	
		36-44	172 (27.3)		0.99 (0.87-1.12)		0.99 (0.93-1.16)	
		45-54	173 (27.4)		1.01 (0.89-1.14)		1.08 (0.89-1.14)	
		≥55	178 (28.2)		1.01 (0.89-1.14)		1.11 (0.95-1.29)	
	**Sex**							
		Female	558 (88.9)		1.00 (reference)		1.00 (reference)	
		Male	70 (11.1)		0.94 (0.82-1.08)		0.95 (0.82-1.11)	
	**Educational level**							
		Below university	205 (32.5)		1.00 (reference)		1.00 (reference)	
		University	426 (67.5)		*1.09 (1.01-1.19)^f^*		*1.19 (1.07-1.32)^g^*	
	**Employment status**							
		Not working	237 (40.2)		1.00 (reference)		1.00 (reference)	
		Working	352 (59.8)		1.03 (0.95-1.13)		1.02 (0.92-1.13)	
	**In a relationship or partnered**							
		No	168 (26.9)		1.00 (reference)		1.00 (reference)	
		Yes	456 (73.1)		*1.14 (1.03-1.26)^f^*		*1.12 (1.01-1.25)^f^*	
	**Country of residence**							
		Australia or New Zealand	187 (29.6)		1.00 (reference)		1.00 (reference)	
		United States or Canada	203 (32.2)		*0.90 (0.81-0.99)^f^*		0.93 (0.83-1.04)	
		United Kingdom	92 (14.6)		1.03 (0.92-1.15)		1.03 (0.92-1.15)	
		Other	149 (23.6)		*0.85 (0.76-0.95)^g^*		*0.84 (0.74-0.96)^g^*	
	**BMI**							
		Underweight or normal	325 (51.6)		1.00 (1.00-1.00)		1.00 (1.00-1.00)	
		Overweight	149 (23.7)		0.92 (0.83-1.02)		0.91 (0.81-1.03)	
		Obese	156 (24.8)		0.93 (0.84-1.03)		0.93 (0.82-1.05)	
	**Perceived support**							
		Lowest	155 (24.6)		1.00 (reference)		1.00 (reference)	
		Second quartile	165 (26.1)		1.06 (0.94-1.19)		0.99 (0.87-1.14)	
		Third quartile	147 (23.3)		1.06 (0.93-1.19)		1.03 (0.90-1.18)	
		Fourth quartile	164 (26)		1.10 (0.98-1.23)		1.11 (0.98-1.25)	
**Health^h^**								
	**MS^i^ type**							
		Nonprogressive	467 (78.9)		1.00 (reference)		1.00 (reference)	
		Progressive	125 (21.1)		0.94 (0.84-1.04)		0.89 (0.78-1.02)	
	**MS duration since diagnosis (years)**							
		≤2	180 (28.5)		1.00 (reference)		1.00 (reference)	
		3-6	158 (25)		1.06 (0.95-1.18)		1.03 (0.91-1.17)	
		7-15	157 (24.9)		0.99 (0.89-1.11)		0.97 (0.85-1.10)	
		>15	136 (21.6)		0.95 (0.85-1.07)		0.92 (0.78-1.07)	
	**Disability (PDDS^j^)**							
		None or mild	338 (53.6)		1.00 (reference)		1.00 (reference)	
		Moderate	231 (36.6)		0.95 (0.87-1.04)		0.98 (0.87-1.05)	
		Severe	62 (9.8)		0.97 (0.84-1.11)		1.00 (0.84-1.18)	
	**Mental quality of life**							
		Lowest	142 (22.5)		1.00 (reference)		1.00 (reference)	
		Second quartile	154 (24.4)		1.08 (0.96-1.23)		1.08 (0.96-1.23)	
		Third quartile	159 (25.2)		1.09 (0.96-1.23)		1.09 (0.96-1.23)	
		Fourth quartile	176 (27.9)		*1.25 (1.11-1.39)^g^*		*1.20 (1.04-1.38)^f^*	
	**Physical quality of life**							
		Lowest	150 (24.3)		1.00 (reference)		1.00 (reference)	
		Second quartile	145 (23.5)		1.08 (0.95-1.23)		1.13 (0.98-1.32)	
		Third quartile	155 (25.1)		*1.13 (1.01-1.28)^f^*		1.14 (0.98-1.33)	
		Fourth quartile	167 (27.1)		*1.21 (1.08-1.36)^g^*		*1.22 (1.04-1.43)^f^*	
	**Self-efficacy**							
		Lowest	170 (27)		1.00 (reference)		1.00 (reference)	
		Second quartile	167 (26.6)		1.10 (0.99-1.23)		1.10 (0.99-1.23)	
		Third quartile	138 (21.9)		1.07 (0.95-1.20)		1.07 (0.95-1.20)	
		Fourth quartile	154 (24.5)		1.05 (0.94-1.18)		1.05 (0.94-1.18)	
	**Fatigue (FSS^k^ >5)**							
		No	297 (47.1)		1.00 (reference)		1.00 (reference)	
		Yes	334 (52.9)		*0.90 (0.84-0.98)^f^*		*0.90 (0.82-0.99)^f^*	
	**Depression**							
		Normal (HADS-D^l^ 0-7)	396 (63)		1.00 (reference)		1.00 (reference)	
		Borderline (HADS-D >7-10)	137 (21.8)		0.92 (0.83-1.02)		0.93 (0.82-1.05)	
		Severe (HADS-D >10-14)	96 (15.3)		*0.85 (0.75-0.96)^f^*		0.86 (0.73-1.00)	
	**Anxiety**							
		Normal (HADS-A^m^ 0-7)	292 (46.3)		1.00 (reference)		1.00 (reference)	
		Borderline (HADS-A >7-10)	143 (22.7)		0.92 (0.83-1.02)		0.90 (0.79-1.02)	
		Severe (HADS-A >10-14)	195 (31)		0.94 (0.86-1.03)		0.97 (0.86-1.08)	
	**Comorbidities (≥1)**							
		No	255 (41.7)		1.00 (reference)		1.00 (reference)	
		Yes	356 (58.3)		0.99 (0.91-1.07)		0.96 (0.87-1.05)	
	**Taking DMTs^n^**							
		No	195 (30.9)		1.00 (reference)		1.00 (reference)	
		Yes	436 (69.1)		1.06 (0.97-1.16)		1.08 (0.97-1.21)	
**Lifestyle related^o^**								
	**Participating in a lifestyle program**							
		No	465 (73.7)		1.00 (reference)		1.00 (reference)	
		Yes	166 (26.3)		0.99 (0.90-1.09)		0.97 (0.88-1.08)	
	**Following a diet program**							
		No	447 (70.8)		1.00 (reference)		1.00 (reference)	
		Yes	184 (29.2)		1.05 (0.97-1.15)		1.06 (0.97-1.17)	
	**Diet quality**							
		Lowest	140 (22.2)		1.00 (reference)		1.00 (reference)	
		Second quartile	160 (25.4)		1.10 (0.97-1.25)		1.08 (0.94-1.25)	
		Third quartile	173 (27.4)		1.11 (0.99-1.26)		1.09 (0.95-1.26)	
		Fourth quartile	158 (25)		*1.21 (1.07-1.36)^f^*		1.14 (0.99-1.31)	
	**Vitamin D supplements (any)**							
		No	90 (14.3)		1.00 (reference)		1.00 (reference)	
		Yes	541 (85.7)		1.13 (1.00-1.29)		1.17 (1.00-1.38)	
	**Omega-3 supplements (any)**							
		No	406 (64.3)		1.00 (reference)		1.00 (reference)	
		Yes	225 (35.7)		1.05 (0.97-1.14)		1.09 (0.99-1.20)	
	**Smoking**							
		Never smoker	359 (56.9)		1.00 (reference)		1.00 (reference)	
		Ex-smoker	223 (35.3)		1.01 (0.93-1.10)		1.01 (0.91-1.10)	
		Current smoker	49 (7.8)		*0.82 (0.69-0.99)^f^*		0.88 (0.72-1.09)	
	**Alcohol consumption**							
		None	134 (23.5)		1.00 (reference)		1.00 (reference)	
		Limited	409 (71.6)		*1.13 (1.02-1.25)^f^*		1.11 (0.99-1.24)	
		Heavy	28 (4.9)		1.13 (0.93-1.38)		1.11 (0.88-1.39)	
	**Meditation**							
		No	450 (71.3)		1.00 (reference)		1.00 (reference)	
		Yes	181 (28.7)		1.02 (0.94-1.12)		1.01 (0.91-1.11)	
	**Exercise (IPAQ^p^)**							
		Inactive	228 (36.1)		1.00 (reference)		1.00 (reference)	
		Minimally active	282 (44.7)		1.05 (0.96-1.15)		1.04 (0.93-1.15)	
		Active	121 (19.2)		1.03 (0.92-1.16)		0.95 (0.82-1.11)	

^a^Adjusted log-binomial regression models for age, sex, educational level, multiple sclerosis type, ongoing symptoms due to recent relapse, number of comorbidities, disability, use of DMTs, participation in another lifestyle intervention, and adherence to a specific diet. Italicized values denote significant association between characteristics and course commencement.

^b^631 participants commenced the MSOC (299 commenced the intervention course and 332 commenced the standard care course). For some variables, due to data unavailability the number of participants within this subgroup does not add up to 631, however the percentages add up to 100%.

^c^PR: prevalence ratio.

^d^aPR: adjusted prevalence ratio.

^e^Sociodemographic-related characteristics.

^f^*P*<.05.

^g^*P*<.01.

^h^Characteristics related to participants’ health.

^i^MS: multiple sclerosis.

^j^PDDS: Patient-Determined Disease Steps.

^k^FSS: Fatigue Severity Scale.

^l^HADS-D: Hospital Anxiety and Depression Scale for symptoms of depression.

^m^HADS-A: Hospital Anxiety and Depression Scale for symptoms of anxiety.

^n^DMT: disease-modifying therapy.

^o^Lifestyle-related characteristics.

^p^IPAQ: International Physical Activity Questionnaire.

### Course Completion

Completion rates were similar across study arms (intervention course: 218/299, 72.9%; standard care course: 224/332, 67.5%; Figure 1). However, factors associated with course completion varied between the intervention course (Table 3) and standard care course (Table 4).

Participants aged 45 to 54 years and ≥55 years were 54% (95% CI 6%-123%) and 96% (95% CI 34%-187%) more likely, respectively, to complete the intervention course than participants aged ≤35 years (Table 3). Being male and following a diet program were associated with 27% (95% CI 7%-51%) and 19% (95% CI 1%-40%) higher intervention course completion, respectively. Clinically significant fatigue was associated with 24% (95% CI 3%-48%) higher intervention course completion. Conversely, people living with MS in the top 3 quartiles of self-efficacy had 35% (95% CI 18%-49%), 26% (95% CI 9%-42%), and 27% (95% CI 7%-43%) lower intervention course completion.

Participants in the standard care course study arm who practiced meditation were 20% (95% CI 2%-41%) more likely to complete the standard care course, whereas those who were employed had 22% (95% CI 8%-30%) lower completion (Table 4).

**Table 3:** Associations between sociodemographic, health, and lifestyle-related characteristics and course completion in the intervention course (n=218)^a^.

Characteristics				Participants (n=218)^b^, n (%)		Univariate analysis, PR^c^ (95% CI)		Multivariate analysis, aPR^d^ (95% CI)
**Sociodemographics^e^**								
	**Age (y)**							
		≤35	29 (13.3)		1.00 (reference)		1.00 (reference)	
		36-44	54 (24.8)		1.11 (0.82-1.51)		1.38 (0.94-2.02)	
		45-54	67 (30.7)		*1.39 (1.05-1.85)^f^*		*1.54 (1.06-2.23)^f^*	
		≥55	68 (31.2)		*1.48 (1.12-1.95)^g^*		*1.96 (1.34-2.87)^h^*	
	**Sex**							
		Female	182 (83.5)		1.00 (reference)		1.00 (reference)	
		Male	36 (16.5)		*1.28 (1.09-1.51)^g^*		*1.27 (1.07-1.51)^g^*	
	**Educational level**							
		Below university	62 (28.4)		1.00 (reference)		1.00 (reference)	
		University	156 (71.6)		1.08 (0.91-1.30)		1.14 (0.93-1.40)	
	**Employment status**							
		Not working	83 (41.5)		1.00 (reference)		1.00 (reference)	
		Working	117 (58.5)		0.99 (0.84-1.17)		1.02 (0.84-1.25)	
	**In a relationship or partnered**							
		No	61 (28.2)		1.00 (reference)		1.00 (reference)	
		Yes	155 (71.8)		0.90 (0.76-1.06)		1.05 (0.85-1.30)	
	**Country of residence**							
		Australia or New Zealand	64 (29.4)		1.00 (reference)		1.00 (reference)	
		United States or Canada	66 (30.3)		0.88 (0.73-1.08)		0.85 (0.68-1.08)	
		United Kingdom	33 (15.1)		0.89 (0.70-1.14)		0.98 (0.77-1.25)	
		Other	55 (25.2)		0.91 (0.74-1.12)		0.97 (0.76-1.23)	
	**BMI (kg/m^2^)**							
		Underweight or normal	127 (58.5)		1.00 (1.00-1.00)		1.00 (1.00-1.00)	
		Overweight	48 (22.1)		0.89 (0.73-1.09)		0.86 (0.68-1.10)	
		Obese	42 (19.4)		0.84 (0.67-1.04)		0.95 (0.74-1.22)	
	**Perceived support**							
		Lowest	52 (23.9)		1.00 (reference)		1.00 (reference)	
		Second quartile	61 (28)		1.09 (0.89-1.34)		1.17 (0.91-1.50)	
		Third quartile	54 (24.8)		1.04 (0.83-1.29)		1.21 (0.95-1.56)	
		Fourth quartile	51 (23.4)		0.87 (0.68-1.11)		0.97 (0.74-1.26)	
**Health related^i^**								
	**MS^j^ type**							
		Nonprogressive	159 (77.6)		1.00 (reference)		1.00 (reference)	
		Progressive	46 (22.4)		1.01 (0.84-1.22)		0.94 (0.76-1.18)	
	**MS duration since diagnosis (years)**							
		≤2	64 (29.4)		1.00 (reference)		1.00 (reference)	
		3-6	48 (22)		0.88 (0.69-1.11)		0.88 (0.69-1.11)	
		7-15	53 (24.3)		1.04 (0.84-1.28)		1.04 (0.84-1.28)	
		>15	53 (24.3)		1.13 (0.92-1.38)		1.13 (0.92-1.38)	
	**Disability (PDDS^k^)**							
		None or mild	112 (51.4)		1.00 (reference)		1.00 (reference)	
		Moderate	84 (38.5)		1.11 (0.94-1.31)		1.01 (0.84-1.22)	
		Severe	22 (10.1)		1.23 (0.97-1.55)		0.86 (0.60-1.23)	
	**Mental quality of life**							
		Lowest	44 (20.2)		1.00 (reference)		1.00 (reference)	
		Second quartile	52 (23.9)		1.12 (0.88-1.43)		1.02 (0.78-1.32)	
		Third quartile	54 (24.8)		1.12 (0.88-1.43)		0.95 (0.73-1.24)	
		Fourth quartile	68 (31.2)		1.16 (0.92-1.46)		0.97 (0.75-1.26)	
	**Physical quality of life**							
		Lowest	50 (23.4)		1.00 (reference)		1.00 (reference)	
		Second quartile	52 (24.3)		1.01 (0.81-1.27)		0.93 (0.81-1.27)	
		Third quartile	63 (29.4)		1.11 (0.91-1.37)		1.11 (0.91-1.31)	
		Fourth quartile	49 (22.9)		0.90 (0.70-1.14)		0.77 (0.57-1.04)	
	**Self-efficacy**							
		Lowest	72 (33.2)		1.00 (reference)		1.00 (reference)	
		Second quartile	51 (23.5)		*0.76 (0.62-0.95)^f^*		*0.65 (0.51-0.82)^h^*	
		Third quartile	47 (21.7)		0.86 (0.70-1.06)		*0.74 (0.58-0.91)^g^*	
		Fourth quartile	47 (21.7)		0.86 (0.70-1.06)		*0.73 (0.57-0.93)^f^*	
	**Fatigue (FSS^l^ >5)**							
		No	96 (44.0)		1.00 (reference)		1.00 (reference)	
		Yes	122 (56.0)		1.14 (0.97-1.34)		*1.24 (1.03-1.48)^f^*	
	**Depression**							
		Normal (HADS-D^m^ 0-7)	142 (65.1)		1.00 (reference)		1.00 (reference)	
		Borderline (HADS-D >7-10)	45 (20.6)		0.92 (0.75-1.13)		0.95 (0.75-1.20)	
		Severe (HADS-D >10-14)	31 (14.2)		1.01 (0.81-1.26)		1.02 (0.79-1.33)	
	**Anxiety**							
		Normal (HADS-A^n^0-7)	104 (47.7)		1.00 (reference)		1.00 (reference)	
		Borderline (HADS-A >7-10)	60 (27.5)		0.98 (0.82-1.17)		1.02 (0.81-1.28)	
		Severe (HADS-A >10-14)	54 (24.8)		0.85 (0.70-1.04)		1.01 (0.80-1.29)	
	**Comorbidities (≥1)**							
		No	88 (41.5)		1.00 (reference)		1.00 (reference)	
		Yes	124 (58.5)		0.97 (0.83-1.14)		0.97 (0.83-1.14)	
	**Taking DMTs^o^**							
		No	71 (32.6)		1.00 (reference)		1.00 (reference)	
		Yes	147 (67.4)		1.10 (0.92-1.30)		1.22 (0.99-1.51)	
**Lifestyle related^p^**								
	**Participating in a lifestyle program**							
		No	160 (73.4)		1.00 (reference)		1.00 (reference)	
		Yes	58 (26.6)		1.05 (0.89-1.25)		1.05 (0.89-1.25)	
	**Following a diet program**							
		No	146 (67.0)		1.00 (reference)		1.00 (reference)	
		Yes	72 (33)		*1.18 (1.01-1.38)^f^*		*1.19 (1.01-1.40)^f^*	
	**Diet quality**							
		Lowest	42 (19.3)		1.00 (reference)		1.00 (reference)	
		Second quartile	51 (23.4)		0.98 (0.75-1.28)		1.06 (0.75-1.28)	
		Third quartile	58 (26.6)		1.16 (0.91-1.48)		1.03 (0.91-1.48)	
		Fourth quartile	67 (30.7)		*1.35 (1.08-1.69)^g^*		1.23 (0.94-1.60)	
	**Vitamin D supplements (any)**							
		No	28 (12.8)		1.00 (reference)		1.00 (reference)	
		Yes	190 (87.2)		1.06 (0.83-1.36)		1.05 (0.78-1.39)	
	**Omega-3 supplements (any)**							
		No	126 (57.8)		1.00 (reference)		1.00 (reference)	
		Yes	92 (42.2)		1.03 (0.88-1.20)		1.03 (0.88-1.20)	
	**Smoking**							
		Never smoker	135 (61.9)		1.00 (reference)		1.00 (reference)	
		Ex-smoker	70 (32.1)		0.94 (0.79-1.12)		0.88 (0.73-1.07)	
		Current smoker	13 (6.0)		0.76 (0.52-1.13)		0.83 (0.54-1.28)	
	**Alcohol consumption**							
		None	42 (21.3)		1.00 (reference)		1.00 (reference)	
		Limited	144 (73.1)		0.99 (0.80-1.22)		1.17 (0.92-1.47)	
		Heavy	11 (5.6)		1.04 (0.76-1.43)		1.15 (0.74-1.79)	
	**Meditation**							
		No	146 (67)		1.00 (reference)		1.00 (reference)	
		Yes	72 (33)		1.16 (0.99-1.36)		1.15 (0.96-1.39)	
	**Exercise (IPAQ^q^)**							
		Inactive	76 (34.9)		1.00 (reference)		1.00 (reference)	
		Minimally active	91 (41.7)		1.02 (0.85-1.22)		0.91 (0.74-1.18)	
		Active	51 (23.4)		1.09 (0.89-1.34)		0.94 (0.73-1.22)	

^a^Adjusted log-binomial regression models for age, sex, educational level, multiple sclerosis type, ongoing symptoms due to recent relapse, number of comorbidities, disability, use of DMTs, participation in another lifestyle intervention, and adherence to a specific diet. Italicized values denote significant association between characteristics and course commencement or completion.

^b^218 participants completed the intervention course. Due to data unavailability the number of participants for certain variables (employment status, BMI, relationship status, MS type, comorbidities, physical quality of life, self-efficacy, and alcohol consumption) is <218, however the percentages for these variables add up to 100%.

^c^PR: prevalence ratio.

^d^aPR: adjusted prevalence ratio.

^e^Sociodemographic-related characteristics.

^f^*P*<.05.

^g^*P*<.01.

^h^*P*<.001.

^i^Characteristics related to participants’ health.

^j^MS: multiple sclerosis.

^k^PDDS: Patient-Determined Disease Steps.

^l^FSS: Fatigue Severity Scale.

^m^HADS-D: Hospital Anxiety and Depression Scale for symptoms of depression.

^n^HADS-A: Hospital Anxiety and Depression Scale for symptoms of anxiety.

^o^DMT: disease-modifying therapy.

^p^Lifestyle-related characteristics.

^q^IPAQ: International Physical Activity Questionnaire.

**Table 4:** Associations between sociodemographic, health, and lifestyle-related characteristics and course completion in the standard care course (n=224)^a^.

Characteristics			Participants, (n=224)^b^, n (%)	Univariate analysis, PR^c^ (95% CI)	Multivariate analysis, aPR^d^ (95% CI)
**Sociodemographics^e^**					
	**Age (y)**				
		≤35	35 (15.6)	1.00 (reference)	1.00 (reference)
		36-44	55 (24.6)	1.05 (0.83-1.33)	1.04 (0.78-1.39)
		45-54	67 (29.9)	*1.24 (1.01-1.54)^f^*	1.18 (0.90-1.54)
		≥55	67 (29.9)	1.12 (0.89-1.40)	1.14 (0.86-1.50)
	**Sex**				
		Female	203 (90.6)	1.00 (reference)	1.00 (reference)
		Male	21 (9.4)	1.09 (0.89-1.33)	1.09 (0.85-1.40)
	**Educational level**				
		Below university	77 (34.4)	1.00 (reference)	1.00 (reference)
		University	147 (65.6)	1.03 (0.90-1.19)	1.10 (0.92-1.31)
	**Employment status**				
		Not working	94 (44.8)	1.00 (reference)	1.00 (reference)
		Working	116 (55.2)	*0.79 (0.70-0.90)^g^*	*0.78 (0.70-0.92)^h^*
	**In a relationship or partnered**				
		No	61 (27.6)	1.00 (reference)	1.00 (reference)
		Yes	160 (72.4)	1.01 (0.87-1.17)	0.97 (0.82-1.14)
	**Country of residence**				
		Australia or New Zealand	71 (31.7)	1.00 (reference)	1.00 (reference)
		United States or Canada	73 (32.6)	1.02 (0.86-1.20)	1.01 (0.84-1.23)
		United Kingdom	34 (15.2)	1.16 (0.97-1.39)	1.11 (0.89-1.39)
		Other	46 (20.5)	0.98 (0.81-1.19)	1.02 (0.79-1.32)
	**BMI (kg/m^2^)**				
		Underweight or normal	106 (47.3)	1.00 (reference)	1.00 (reference)
		Overweight	55 (24.6)	1.03 (0.88-1.21)	1.04 (0.85-1.28)
		Obese	63 (28.1)	1.01 (0.86-1.18)	0.98 (0.80-1.21)
	**Perceived support**				
		Lowest	59 (26.3)	1.00 (reference)	1.00 (reference)
		Second quartile	61 (27.2)	0.98 (0.83-1.17)	1.02 (0.83-1.26)
		Third quartile	51 (22.8)	0.97 (0.80-1.16)	0.99 (0.80-1.24)
		Fourth quartile	53 (23.7)	0.91 (0.75-1.10)	0.98 (0.78-1.23)
**Health related^i^**					
	**MS^j^ type**				
		Nonprogressive	166 (78.3)	1.00 (reference)	1.00 (reference)
		Progressive	46 (21.7)	1.12 (0.97-1.29)	1.07 (0.88-1.31)
	**MS duration since diagnosis (years)**				
		≤2	61 (27.2)	1.00 (reference)	1.00 (reference)
		3-6	59 (26.3)	1.07 (0.90-1.27)	1.07 (0.90-1.27)
		7-15	54 (24.1)	0.90 (0.75-1.12)	0.90 (0.75-1.12)
		>15	50 (22.3)	1.05 (0.88-1.26)	1.05 (0.88-1.26)
	**Disability (PDDS^k^)**				
		None or mild	116 (51.8)	1.00 (reference)	1.00 (reference)
		Moderate	87 (38.8)	1.08 (0.95-1.23)	1.08 (0.90-1.30)
		Severe	21 (9.4)	0.86 (0.65-1.13)	0.83 (0.59-1.16)
	**Mental quality of life**				
		Lowest	52 (23.2)	1.00 (reference)	1.00 (reference)
		Second quartile	54 (24.1)	0.93 (0.76-1.13)	0.96 (0.78-1.18)
		Third quartile	57 (25.4)	0.96 (0.79-1.15)	0.78 (0.61-1.01)
		Fourth quartile	61 (27.2)	1.04 (0.87-1.23)	1.02 (0.83-1.25)
	**Physical quality of life**				
		Lowest	55 (25.1)	1.00 (reference)	1.00 (reference)
		Second quartile	47 (21.5)	0.94 (0.77-1.16)	0.92 (0.71-1.19)
		Third quartile	52 (23.7)	1.01 (0.84-1.23)	0.98 (0.77-1.24)
		Fourth quartile	65 (29.7)	1.04 (0.87-1.24)	1.01 (0.77-1.35)
	**Self-efficacy**				
		Lowest	51 (22.8)	1.00 (reference)	1.00 (reference)
		Second quartile	59 (26.3)	1.10 (0.90-1.34)	1.07 (0.83-1.38)
		Third quartile	52 (23.2)	1.16 (0.95-1.41)	1.12 (0.88-1.44)
		Fourth quartile	62 (27.7)	1.11 (0.91-1.35)	1.09 (0.84-1.41)
	**Fatigue (FSS^l^ >5)**				
		No	106 (47.3)	1.00 (reference)	1.00 (reference)
		Yes	118 (52.7)	0.98 (0.86-1.12)	1.08 (0.91-1.27)
	**Depression**				
		Normal (HADS-D^m^ 0-7)	138 (61.9)	1.00 (reference)	1.00 (reference)
		Borderline (HADS-D >7-10)	47 (21.1)	0.97 (0.82-1.15)	1.01 (0.83-1.22)
		Severe (HADS-D >10-14)	38 (17.0)	1.01 (0.84-1.20)	0.96 (0.76-1.22)
	**Anxiety**				
		Normal (HADS-A^n^ 0-7)	105 (46.9)	1.00 (reference)	1.00 (reference)
		Borderline (HADS-A >7-10)	43 (19.2)	1.07 (0.91-1.26)	1.05 (0.85-1.30)
		Severe (HADS-A >10-14)	76 (33.9)	0.99 (0.85-1.15)	0.95 (0.78-1.15)
	**Comorbidities (≥1)**				
		No	90 (42.1)	1.00 (reference)	1.00 (reference)
		Yes	124 (57.9)	1.03 (0.90-1.19)	1.03 (0.88-1.21)
	**Taking DMTs^o^**				
		No	65 (29.0)	1.00 (reference)	1.00 (reference)
		Yes	159 (71.0)	0.89 (0.78-1.02)	0.84 (0.70-1.00)
**Lifestyle related^p^**					
	**Participating in a lifestyle program**				
		No	164 (73.2)	1.00 (reference)	1.00 (reference)
		Yes	60 (26.8)	0.98 (0.85-1.14)	0.98 (0.85-1.14)
	**Following a diet program**				
		No	161 (71.9)	1.00 (reference)	1.00 (reference)
		Yes	63 (28.1)	0.97 (0.83-1.12)	0.93 (0.78-1.12)
	**Diet quality**				
		Lowest	45 (20.1)	1.00 (reference)	1.00 (reference)
		Second quartile	57 (25.4)	1.21 (0.99-1.49)	1.24 (0.95-1.63)
		Third quartile	64 (28.6)	1.11 (0.90-1.37)	1.15 (0.87-1.52)
		Fourth quartile	58 (25.9)	1.20 (0.98-1.48)	1.19 (0.90-1.58)
	**Vitamin D supplements (any)**				
		No	31 (13.8)	1.00 (reference)	1.00 (reference)
		Yes	193 (86.2)	1.10 (0.90-1.36)	1.04 (0.82-1.33)
	**Omega-3 supplements (any)**				
		No	161 (71.9)	1.00 (reference)	1.00 (reference)
		Yes	63 (28.1)	0.95 (0.82-1.11)	0.95 (0.82-1.11)
	**Smoking**				
		Never smoker	125 (55.8)	1.00 (reference)	1.00 (reference)
		Ex-smoker	80 (35.7)	0.90 (0.78-1.05)	0.91 (0.77-1.07)
		Current smoker	19 (8.5)	1.02 (0.82-1.27)	1.07 (0.82-1.38)
	**Alcohol consumption**				
		None	45 (22.1)	1.00 (reference)	1.00 (reference)
		Limited	148 (72.6)	1.01 (0.85-1.20)	1.19 (0.95-1.47)
		Heavy	11 (5.4)	1.15 (0.87-1.51)	1.27 (0.89-1.83)
	**Meditation**				
		No	156 (69.6)	1.00 (reference)	1.00 (reference)
		Yes	68 (30.4)	*1.15 (1.01-1.31)^f^*	*1.20 (1.02-1.41)^f^*
	**Exercise (IPAQ^q^)**				
		Inactive	77 (34.4)	1.00 (reference)	1.00 (reference)
		Minimally active	109 (48.7)	1.09 (0.93-1.26)	1.15 (0.95-1.40)
		Active	38 (17.0)	1.12 (0.93-1.35)	1.25 (0.97-1.62)

^a^Adjusted log-binomial regression models for age, sex, educational level, multiple sclerosis type, ongoing symptoms due to recent relapse, number of comorbidities, disability, use of DMTs, participation in another lifestyle intervention, and adherence to a specific diet. Italicized values denote significant association between characteristics and course commencement or completion.

^b^224 participants completed the standard care course. Due to data inavailability the number of participants for certain variables (employment status, relationship status, MS type, depression, comorbidities, physical quality of life, self-efficacy, and alcohol consumption) is <224, however the percentages for these variables add up to 100%.

^c^PR: prevalence ratio.

^d^aPR: adjusted prevalence ratio.

^e^Sociodemographic-related characteristics.

^f^*P*<.05.

^g^*P*<.01.

^h^*P*<.001.

^i^Characteristics related to participants’ health.

^j^MS: multiple sclerosis.

^k^PDDS: Patient-Determined Disease Steps.

^l^FSS: Fatigue Severity Scale.

^m^HADS-D: Hospital Anxiety and Depression Scale for symptoms of depression.

^n^HADS-A: Hospital Anxiety and Depression Scale for symptoms of anxiety.

^o^DMT: disease-modifying therapy.

^p^Lifestyle-related characteristics.

^q^IPAQ: International Physical Activity Questionnaire.

## Discussion

### Principal Findings

Our study examined the rates of completion and commencement of a web-based course on modification of lifestyle-related risk factors for people living with MS. We found that conversion rates from initial enrollment to course commencement were relatively low (631/1893, 33.33%). However, commencement rates in those who had completed the baseline survey after enrollment were relatively high (631/857, 73.6%), as were completion rates for those who had commenced the course (218/444, 49.1% for the intervention course and 224/413, 54.2% for the standard care course). Our study also sheds light on the factors associated with course commencement and completion. Those factors related to course commencement of potentially practical interest included educational level, being in a relationship, and clinical factors such as QoL and fatigue. Factors of potential interest associated with completion included age of >45 years, male sex, being employed, self-efficacy, and already having modified some risk factors such as diet and undertaking stress-reducing activities before course commencement. We also acknowledge that the length of the baseline survey (166 questions) may have influenced commencement and completion rates, with more motivated participants and possibly more able people likely to have both commenced and completed the MSOC.

The 166-question baseline survey was estimated to take 45 to 60 minutes, and completion was requested (but not mandated) before commencing the course. This may have posed a barrier for some individuals as only 45.27% (857/1893) of participants enrolled in the MSOC effectiveness RCT completed the survey. Data are not available as to how many participants started but did not complete the survey (ie, those that attempted the survey but “gave up” before commencing the course). In hindsight, a qualitative analysis of people who did not complete the baseline survey would have been beneficial to determine the influence that the baseline survey had on MSOC commencement and completion rates and is a strong recommendation for future studies.

Notably, low rates of baseline survey completion are consistent with our previous MSOC feasibility RCT (42%) [34]. This may be attributed to time constraints or disease-related limitations, including visual impairment or cognitive fatigue. The baseline survey was adapted from the survey used in the Health Outcomes and Lifestyle in a Sample of People With Multiple Sclerosis study conducted by this research group [51]. In this study, the extensive nature of the survey enabled prospective analyses of associations between lifestyle-related risk factors and health outcomes. Despite substantial participant attrition over 7.5 years of follow-up, significant associations were found between lifestyle modification and improved health outcomes [3,10]. Hence, the research team, while understanding that the survey may be a barrier to completion, elected to use the baseline survey to obtain sufficient data to reach robust conclusions regarding the effect of the MSOC on QoL and health outcomes into the future. Future researchers need to make informed choices regarding the length of data collection surveys and their impact on course commencement and completion and weigh the potential benefits of increased data against the attrition and potential bias related to noncommencement. Future studies could consider the use of a shorter adaptive questionnaire to possibly enhance participation in the context of an RCT.

We also found positive associations between course commencement and a university degree, consistent with reported findings of associations between higher education and positive attitudes toward health research and a willingness to participate [52,53]. People with higher educational levels may have had more exposure to, experience of, and capabilities with using digital technology. Those with higher educational levels may possibly have found the baseline survey less onerous due to enhanced digital skills and, therefore, less of a barrier to commencement. However, higher education was not associated with course completion.

Participants in a relationship or with higher perceived support were more likely to commence the MSOC, consistent with reported associations between supportive relationships and digital intervention engagement [54]. Furthermore, support from family, teachers, and friends that students receive, as well as organizational support, is related to increased adherence to web-based learning [55,56]. MSOC participants received organizational support provided by a full-time course coordinator whom they were able to directly email for advice and support with technical issues. They were also provided with support from peers and facilitators in the forum who would answer direct questions regarding course content and facilitate discussions. These factors may explain the association between perceived support and MSOC commencement, as well as the high rates of intervention course and standard care course completion among course commencers.

Consistent with studies highlighting the importance of health and well-being in digital health engagement [57,58], participants with higher QoL (ie, greater health and well-being) were more likely to commence the MSOC, whereas higher fatigue was associated with decreased MSOC commencement. Similarly, a recent systematic review of digital health intervention engagement found that lower health-related QoL and higher depression were significant barriers to engagement [59]. Given that improving QoL is a central aim of both this course and similar lifestyle modification programs, it is imperative for future research to identify and address the unmet needs of people living with MS who report lower QoL as they are likely to derive the greatest benefit from web-based interventions.

Intervention course and standard care course completion rates were 49.1% (218/444) and 54.2% (224/413), respectively, comparable to 60% (9/15) and 50% (8/16) in the MSOC feasibility RCT [34]. Similarly, Claflin et al [29] reported *Understanding MS* MOOC completion rates of 42%. In contrast, a recent meta-analysis of completion rates by people living with MS across 32 RCTs reported considerably lower completion rates (15%-17%) [28]. It is possible that the duration of the digital health program may affect completion rates as 28 of the 32 web-based programs in the meta-analysis spanned 8 to 52 weeks, whereas both the MSOC and the *Understanding MS* MOOC were 6-week programs.

Participants aged >45 years had higher intervention course completion. This was similar to other studies that found that middle-aged people were most likely to complete web-based lifestyle-related health interventions [54,60]. A meta-analysis of 10 RCTs examining engagement in web-based interventions for depression identified that younger age is associated with higher dropout rates and rates of completion also seem to decrease among individuals aged >65 years [61]. This phenomenon has been ascribed to various factors, including digital proficiency, physical limitations, and lack of interest [62].

Interestingly, being male was associated with higher intervention course completion, which is different from the meta-analysis findings, where the greatest dropout from web-based interventions was found among male individuals [61]. Despite only 9.4% (21/224) and 16.5% (36/218) of participants in the standard care course and intervention course being male, respectively, these proportions reflect the rates of MS diagnoses, as considerably more female than male individuals are diagnosed with MS, and are similar to other well-studied MS cohorts [63]. Moreover, it is important to highlight the small number of male individuals in our cohort, so caution needs to be taken when interpreting these study findings.

Employed participants had 22% lower standard care course completion, which was similar to a prospective pilot study that identified links between work-life balance issues and lower completion of a 15-day web-based well-being intervention [64]. Similarly, time constraints from other life responsibilities were a major reason for noncompletion of the *Understanding MS* MOOC by people living with MS [65]. However, it is not clear why employment was associated with lower standard care course completion but not intervention course completion. Perhaps participants were more engaged with the novel content of the intervention course compared with that of the standard care course, or other factors such as the greater engagement with the community forum observed for intervention course versus standard care course participants may have played a role in greater intervention course completion among employed participants. Nevertheless, as time limitations are a commonly reported reason for noncompletion of web-based interventions [64,65], appropriate measures, such as increased time to complete web-based courses, could be implemented to increase engagement by people living with MS, especially those who are employed. Furthermore, many participants who were interviewed a month after course completion mentioned that they found it difficult to find time to complete the course, with some commenting that more user-friendly platforms such as mobile devices rather than sitting at a computer would be helpful (data not shown).

Greater self-efficacy was found to be associated with lower intervention course completion. This was unexpected considering that self-efficacy is associated with commencement and maintenance of healthy behaviors, including healthy eating, physical activity, and smoking cessation [66], and increased engagement with web-based lifestyle interventions [67]. The quantitative impact of completing the intervention course on self-efficacy levels remains to be assessed. However, qualitative insights from intervention course participants who completed it suggested that participants sought information for the purposes of enhancing self-management [57], resulting in perceived improvements in self-efficacy [58]. One possible explanation is that participants with higher baseline self-efficacy levels felt necessarily equipped to independently initiate and sustain lifestyle change, whereas others persisted to improve self-efficacy and were reinforced by noticeable improvements in self-efficacy.

Participants adhering to an MS-specific diet program had a 19% higher intervention course completion, and those practicing meditation had 20% higher standard care course completion. While not consistent across study arms, these results may suggest that people living with MS adopting a healthy lifestyle may have a greater interest or desire to learn lifestyle-related knowledge and, therefore, were more likely to complete the web-based course. Conversely, participants with a less healthy lifestyle or those lacking motivation have been shown to lose interest in multimodal lifestyle interventions [68].

### Suggestions for Future Research

Our findings underscore the importance of considering individual differences in end-user characteristics when developing and delivering web-based interventions, which may be relevant in the development of interventions for other chronic illnesses. In particular, we found that people aged >45 years were more likely to complete the web-based intervention, emphasizing the importance of developing programs that are appropriate in format (eg, videos and text) and content across different ages. As we found that higher social support was associated with increased commencement, consistent with other studies [54], providing greater support for end users may increase commencement and adherence to web-based interventions, for instance, including a facilitator-run community forum component nested within the intervention, as with the MSOC. Similarly, our findings (associations between reduced QoL and reduced commencement), in line with a recent systematic review [59], emphasize the need to consider the health and well-being of the end-user population to increase engagement. While this may be difficult to address, including trained support staff such as mental health professionals or clinicians may help participants seek help or resources, which in turn may have the cascade effect of both improving well-being and increasing engagement. This is especially relevant for people living with MS and other chronic conditions such as cardiovascular disease and cancer due to the high prevalence of depression and anxiety among these populations [69-71]. Importantly, our findings highlight the need for future studies to examine strategies to overcome factors influencing low commencement and adherence.

### Strengths and Limitations

Technological elements (eg, interactivity and multimedia components) of digital engagement by people living with MS have previously been examined through systematic reviews and meta-analyses [28,30], and barriers to MOOC participation were recently evaluated in a mixed methods study [65]. However, this study provides an extensive evaluation of the relationship between participant characteristics and the different stages of MSOC engagement and of the factors influencing the commencement and completion of a web-based lifestyle modification course across both study arms, which has not been previously reported.

However, there are study limitations that need to be outlined. First, as the results are based on self-reported baseline data, there is the potential for recall bias. Second, the possibility of selection bias exists as participants were possibly more motivated or interested in learning about lifestyle-related risk factors than the general MS population. Third, the generalizability of the study findings may be further limited as the study cohort specifically comprised individuals who had completed the extensive 166-question baseline survey, indicating that they were likely to be even more highly motivated than the general MS population. Fourth, the cohort were necessarily English speaking and predominantly resided in Westernized countries, so cultural differences could not be explored due to the limited sample sizes from other countries. Fifth, some of the baseline characteristics, such as meditation practice, were queried in a dichotomous manner, which may reduce the usefulness of the study findings and their implications. However, certain questions had to be restricted to avoid questionnaire burden and potentially increase study dropout. Sixth, the adjusted analysis may not control for all potential confounders, and the dichotomous nature of some of the variables could mean that adjustment was incomplete. Consequently, it is important to note that confounding may still be present. As a follow-up to this, as our results represent an exploratory analysis complementary to the primary and secondary aims of the ancillary RCT, we advise caution when interpreting the study findings given the potential for incomplete adjustment. This is especially relevant for significant variables for which the lower boundary of the 95% CI of the adjusted prevalence ratio was close to 1.

### Conclusions

This study identified specific participant characteristics associated with different stages of MSOC engagement. Factors associated with course commencement included a university education and having greater perceived support and greater mental and physical QoL. Factors associated with course completion included older age, being male, and adherence to a diet program. Improved commencement rates may be obtained with shorter initial data collection surveys to decrease potential barriers to commencement depending on the aims of the study. Other potential interventions to enhance completion include ensuring adequate time for completion to address fatigue and health-related issues and the provision of technical support to participants throughout the RCT. Involving other people such as family members to complete web-based learning programs alongside people living with MS could also provide further support and facilitate course completion. Collectively, the study findings provide practical considerations for the future design, development, and implementation of digital lifestyle interventions for people living with MS. The findings also highlight the need for further quantitative and qualitative studies to provide greater depth of understanding into digital health engagement by people living with MS.

## Appendix

Multimedia Appendix 1 CONSORT-eHEALTH checklist (V 1.6.1).

Multimedia Appendix 2 Outline of the intervention and standard care course format.

Multimedia Appendix 3 Participants’ country of residence.

Multimedia Appendix 4 Disease-modifying and prescription medications taken by Multiple Sclerosis Online Course participants.

## Abbreviations

CONSORT-R: Consolidated Standards of Reporting Trials–Routine
DHQ: Diet Habits Questionnaire
DMT: disease-modifying therapy
IPAQ: International Physical Activity Questionnaire
MOOC: massive open online course
MS: multiple sclerosis
MSOC: Multiple Sclerosis Online Course
OMS: Overcoming Multiple Sclerosis
QoL: quality of life
RCT: randomized controlled trial
UWSES: University of Washington Self-Efficacy Scale

## Data Availability

The data set generated and analyzed during this study are in a reidentifiable format. This data set is not publicly due to the study currently being in progress but the data set will be available from the corresponding author on reasonable request at the completion of the study.
