# Supplementary material for: Sociodemographic, Health, and Lifestyle-Related Characteristics Associated With the Commencement and Completion of a Web-Based Lifestyle Educational Program for People With Multiple Sclerosis: Randomized Controlled Trial
Source: J Med Internet Res. 2024 Aug 28;26:e58253. doi: 10.2196/58253 (PMC11391162; doi:10.2196/58253)
Supplement: Multimedia Appendix 2 [file jmir_v26i1e58253_app2.docx]

**Multimedia Appendix 2.** Outline of intervention and standard-care course format

| **Week** | **Modules** | **Intervention** | **Standard-care** |
| --- | --- | --- | --- |
| 1 | Introduction | Introductions of course practitioners and tutorial on course functionalities, format and timing | |
|  | Diet and supplementation | A plant-based wholefood diet plus seafood, with very low saturated fat (<20g/day); no dairy, meat, palm, or coconut oil. Omega-3 fatty acid supplement use (20-40ml per day). Alcohol consumption follow national guidelines | Eat a balanced diet and follow national guidelines. Common diets: gluten free, Paleolithic, McDougal, and Mediterranean diets. Alcohol consumption follow national guidelines |
| 2 | Vitamin D and sunlight; | Sunlight: 15min/day UV index 7 (or equivalent) 3-5 times/week; or Vitamin D3 supplement use ≥5000IU/day | No general recommendations. Up to 2000IU supplement for deficiency. |
|  | Physical activity | 20-30 mins, 5 times/week physical activity | 30 mins moderate aerobic activity and strength training 2 times/week |
| 3 | Stress reduction | 30 min/day meditation/ mindfulness/stress-reducing activities | No conclusive evidence (no information provided) |
|  | Family and prevention | Genetic risk of getting MS and prevention via lifestyle; no smoking; role of medication | Genetic risk of getting MS, smoking increases risk of MS; role of medication |
| 4 | Concluding remarks | Concluding remarks and closing ceremony | |
| 5/6 | Catch-up |  | |
