# Supplementary material for: Sociodemographic, Health, and Lifestyle-Related Characteristics Associated With the Commencement and Completion of a Web-Based Lifestyle Educational Program for People With Multiple Sclerosis: Randomized Controlled Trial
Source: J Med Internet Res. 2024 Aug 28;26:e58253. doi: 10.2196/58253 (PMC11391162; doi:10.2196/58253)
Supplement: Multimedia Appendix 3 [file jmir_v26i1e58253_app3.docx]

**Multimedia Appendix 3.** Country of residence

| **Country of residence** | **N (%)** |
| --- | --- |
| Albania | 1 (0.1) |
| Algeria | 1 (0.1) |
| Argentina | 3 (0.4) |
| Armenia | 1 (0.1) |
| Australia | 206 (24) |
| Austria | 4 (0.5) |
| Azerbaijan | 1 (0.1) |
| Belgium | 1 (0.1) |
| Bosnia and Herzegovina | 3 (0.4) |
| Brazil | 1 (0.1) |
| Bulgaria | 8 (0.9) |
| Canada | 106 (12.4) |
| China | 1 (0.1) |
| Croatia | 10 (1.2) |
| Cyprus | 1 (0.1) |
| Czech Republic | 19 (2.2) |
| Estonia | 8 (0.9) |
| Finland | 2 (0.2) |
| France | 11 (1.3) |
| Germany | 13 (1.5) |
| Greece | 10 (1.2) |
| India | 3 (0.4) |
| Iran | 3 (0.4) |
| Ireland | 12 (1.4) |
| Israel | 1 (0.1) |
| Italy | 9 (1.1) |
| Japan | 1 (0.1) |
| Jordan | 2 (0.2) |
| Kazakhstan | 2 (0.2) |
| Latvia | 1 (0.1) |
| Lithuania | 1 (0.1) |
| Mexico | 9 (1.1) |
| Montenegro | 1 (0.1) |
| Morocco | 3 (0.4) |
| Netherlands | 6 (0.7) |
| New Zealand | 30 (3.5) |
| Norway | 2 (0.2) |
| Pakistan | 1 (0.1) |
| Poland | 7 (0.8) |
| Portugal | 1 (0.1) |
| Romania | 1 (0.1) |
| Russian Federation | 11 (1.3) |
| Serbia | 7 (0.8) |
| South Africa | 10 (1.2) |
| Spain | 3 (0.4) |
| Sweden | 2 (0.2) |
| Switzerland | 1 (0.1) |
| Syria | 1 (0.1) |
| Taiwan | 3 (0.4) |
| Ukraine | 18 (2.1) |
| United Arab Emirates | 1 (0.1) |
| United Kingdom | 113 (13.2) |
| United States | 180 (21.0) |
| **Total** | **857 (100.0)** |
