# Supplementary material for: Sociodemographic, Health, and Lifestyle-Related Characteristics Associated With the Commencement and Completion of a Web-Based Lifestyle Educational Program for People With Multiple Sclerosis: Randomized Controlled Trial
Source: J Med Internet Res. 2024 Aug 28;26:e58253. doi: 10.2196/58253 (PMC11391162; doi:10.2196/58253)
Supplement: Multimedia Appendix 4 [file jmir_v26i1e58253_app4.docx]

**Multimedia Appendix 4.** Disease-modifying and prescription medications taken by MSOC participants

| **Disease-Modifying Treatment** | **N (%)** |
| --- | --- |
| Adrenocorticotropic hormone (ACTH, Acthar®) |  |
| No | 857 (100.0%) |
| Yes | 0 (0%) |
| Alemtuzumab (Campath®, Lemtrada®) |  |
| No | 851 (99.3%) |
| Yes | 6 (0.7%) |
| Autologous stem cell transplantation |  |
| No | 855 (99.8%) |
| Yes | 2 (0.2%) |
| Azathioprine (Imuran®, Azasan®) |  |
| No | 855 (99.8%) |
| Yes | 2 (0.2%) |
| Cladribine (Leustat,  Movectro, Movectra) |  |
| No | 829 (96.7%) |
| Yes | 28 (3.3%) |
| Cyclophosphamide  (Cytoxan,  Revimmune) |  |
| No | 856 (99.9%) |
| Yes | 1 (0.1%) |
| Daclizumab (Zenapax®) |  |
| No | 857 (100.0%) |
| Diroximel fumarate (Vumerity®) |  |
| No | 852 (99.4%) |
| Yes | 5 (0.6%) |
| Fampridine (Fampyra®, Ampyra®) |  |
| No | 834 (97.3%) |
| Yes | 23 (2.7%) |
| Fingolimod (FTY-720, Gilenya®) |  |
| No | 823 (96.0%) |
| Yes | 34 (4.0%) |
| Glatiramer Acetate (Copaxone®, Glatopa®) |  |
| No | 812 (94.7%) |
| Yes | 45 (5.3%) |
| Interferons (Avonex®, Betaferon®, Betaseron®, Extavia®, Rebif®, Piegridy®) |  |
| No | 823 (96.0%) |
| Yes | 34 (4.0%) |
| Laquinimod (Nerventra®) |  |
| No | 855 (99.8%) |
| Yes | 2 (0.2%) |
| Low-dose Naltrexone(LDN) |  |
| No | 853 (99.5%) |
| Yes | 4 (0.5%) |
| Methotrexate (Folex,  Matrex, Rheumatrex,  Trexall) |  |
| No | 856 (99.9%) |
| Yes | 1 (0.1%) |
| Minocycline(Minomycin) |  |
| No | 856 (99.9%) |
| Yes | 1 (0.1%) |
| Mitoxantrone(Novantrone®) |  |
| No | 857 (100.0%) |
| Monomethyl fumarate(Bafiertaim®) |  |
| No | 857 (100.0%) |
| Mycophenolate Mofetil (Cellcept®) |  |
| No | 856 (99.9%) |
| Yes | 1 (0.1%) |
| Natalizumab (Tysabri®) |  |
| No | 801 (93.5%) |
| Yes | 56 (6.5%) |
| Ocrelizumab (Ocrevus) |  |
| No | 693 (80.9%) |
| Yes | 164 (19.1%) |
| Ofatumumab (Kesimpta®) |  |
| No | 804 (93.8%) |
| Yes | 53 (6.2%) |
| Ozanimod (Zeposia®) |  |
| No | 850 (99.2%) |
| Yes | 7 (0.8%) |
| Peginterferon Beta-1a |  |
| No | 854 (99.6%) |
| Yes | 3 (0.4%) |
| Plasmapheresis/Plasma exchange |  |
| No | 854 (99.6%) |
| Yes | 3 (0.4%) |
| Rituximab (Rituxan®) |  |
| No | 840 (98.0%) |
| Yes | 17 (2.0%) |
| Siponimod (Mayzent®) |  |
| No | 849 (99.1%) |
| Yes | 8 (0.9%) |
| Steroids (Prednisone, Prednisolone) |  |
| No | 844 (98.5%) |
| Yes | 13 (1.5%) |
| Teriflunomide (Aubagio®) |  |
| No | 835 (97.4%) |
| Yes | 22 (2.6%) |
| Other |  |
| No | 798 (93.1%) |
| Yes | 59 (6.9%) |
| **Prescription medicine** | **N (%)** |
| Depression |  |
| No | 665 (77.6%) |
| Yes | 192 (22.4%) |
| Anxiety |  |
| No | 707 (82.5%) |
| Yes | 150 (17.5%) |
| Headaches |  |
| No | 762 (88.9%) |
| Yes | 95 (11.1%) |
| Pain (other than headaches) |  |
| No | 689 (80.4%) |
| Yes | 168 (19.6%) |
| Fatigue |  |
| No | 759 (88.6%) |
| Yes | 98 (11.4%) |
| Difficulty sleeping at night |  |
| No | 742 (86.6%) |
| Yes | 115 (13.4%) |
| Bladder problems |  |
| No | 752 (87.7%) |
| Yes | 105 (12.3%) |
| Bowel problems |  |
| No | 808 (94.3%) |
| Yes | 49 (5.7%) |
| Spasticity |  |
| No | 729 (85.1%) |
| Yes | 128 (14.9%) |
| Other |  |
| No | 759 (88.6%) |
| Yes | 98 (11.4%) |
| **Total** | **857 (100%)** |
